# A 10-year surveillance of Rickettsiales (*Rickettsia* spp. and *Anaplasma phagocytophilum*) in the city of Hanover, Germany, reveals *Rickettsia* spp. as emerging pathogens in ticks

**DOI:** 10.1186/s13071-017-2537-2

**Published:** 2017-11-28

**Authors:** Katrin Blazejak, Elisabeth Janecek, Christina Strube

**Affiliations:** Institute for Parasitology, Centre for Infection Medicine, University of Veterinary Medicine Hannover, Buenteweg 17, 30559 Hannover, Germany

**Keywords:** Tick-borne diseases, *Ixodes ricinus*, *Anaplasma phagocytophilum*, *Rickettsia*, Surveillance, Public health, Epidemiology

## Abstract

**Background:**

Rickettsiales (*Rickettsia* spp. and *Anaplasma phagocytophilum*) transmitted by ticks are considered (re-)emerging pathogens posing a risk to public health. Nevertheless, year-long monitoring studies on prevalences of these pathogens in questing ticks to contribute to public health risk assessment are rare.

**Methods:**

The current study extends previous prevalence surveillances (2005 and 2010) by 2015 to a 10-year monitoring. Therefore, 2100 questing *Ixodes ricinus* were collected from April to October 2015 at ten different recreation sites in the city of Hanover, Germany, to determine potential changes in tick infection rates with Rickettsiales.

**Results:**

Of the collected ticks, 288 were adult females, 285 adult males and 1527 nymphs. Overall, 3.8% (79/2100) of ticks were infected with *A. phagocytophilum*, 50.8% (1066/2100) with *Rickettsia* spp. and 2.2% (46/2100) with both pathogens. Statistical analyses revealed stagnating *A. phagocytophilum* infection rates over the 10-year monitoring period, whereas *Rickettsia* infections increased significantly (33.3% in 2005 and 26.2% in 2010 *vs* 50.8% in 2015). This increase was also characterized by prominent seasonality with higher prevalences from July to October.

**Conclusions:**

As increased tick infection rates result in an increased risk for public health, the long-term data reported here provide significant implications for the understanding of progressing Rickettsiales distribution in ticks and essentially contribute to reliable public health risk assessments.

## Background

Tick-borne diseases may have considerable impact on human and animal health. In central Europe, the widespread castor bean tick *Ixodes ricinus* transmits a variety of bacterial pathogens such as *Anaplasma phagocytophilum* or different *Rickettsia* species of the spotted fever group, which are considered (re-)emerging pathogens [1]. Both microorganisms are classified as Gram-negative, obligate intracellular α-proteobacteria belonging to the order Rickettsiales [2].

To maintain its life cycle, *A. phagocytophilum* is reliant on ticks as vectors and vertebrates like small mammals, sheep or deer as reservoir hosts [3]. In Europe, human pathogenic strains appear to prefer dogs, horses, hedgehogs or wild boars as reservoir hosts, whereas strains occurring in deer seem to be less pathogenic to humans [4]. In humans, granulocytic anaplasmosis is associated with variable symptoms such as myalgia, fever and malaise as well as decreased blood cell counts, thrombocytopenia or leukopenia [5]. In North America, circulating strains of the bacterium may lead to even more severe outcomes as about 3.0% of patients develop life threatening complications and at least 1.0% of infections are fatal [5]. Conversely, case reports of human granulocytic anaplasmosis (HGA) from Europe are sporadic with identification of different strains, resulting in less severe disease [4–7]. However, estimated seroprevalences of antibodies against *A. phagocytophilum* appear to be considerably higher in contrast to prevalences in questing ticks. For example, seroprevalences in tick-exposed individuals in Germany range between 11.4–14.0% [6], whereas the distribution of *A. phagocytophilum* in questing *I. ricinus* varies between 0 and 9.5% [8–13]. In Switzerland, solely 0.4–2.1% of *I. ricinus* are infected; however, seroprevalences in tick-exposed individuals reach up to 17.1%, which is eight times higher than prevalences in ticks [6]. Comparable prevalences in ticks are also described for other European countries [14–16]. In ruminants, *A. phagocytophilum* induced tick-borne fever causes considerable economic losses due to clinical manifestations such as abortion, reduced milk production or sudden death [3].

In contrast to *A. phagocytophilum*, different *Rickettsia* species utilize *I. ricinus* as a vector, main host and reservoir host [17]. To date, six species (*R. slovaca, R. helvetica, R. felis, R. monacensis, R. massiliae* and *R. raoultii*, formerly known as *Rickettsia* sp. RpA4) have been detected in *I. ricinus* or *Dermacentor* spp. in Germany [18]. Of these, four have been detected in *I. ricinus* with *R. helvetica* representing the predominant species [8, 9, 13, 18, 19]. In humans, *Rickettsia* spp. may cause different forms of spotted fever as well as lymphadenopathy or “summer flu”, an unspecific and uneruptive feverish infection [18]. Although *R. helvetica* infections are mainly associated with mild clinical manifestations, potentially life-threatening cases of meningitis or perimyocarditis have been described [20, 21]. Nevertheless, it may be assumed that most cases of *R. helvetica* infections are misdiagnosed due to lacking awareness of rickettsiosis in Europe [22]. Prevalences of *Rickettsia* spp. in *I. ricinus* are widely available [8, 9, 13, 19, 23] with the so far highest prevalence of 52.5% determined in the city of Hamburg, Germany [13]. Regarding humans, Sonnleitner et al. [24] determined 7.7% human blood donors in Tyrol, Austria, as seropositive for *Rickettsia* species.

Distribution of pathogenic microorganisms in ticks should be continuously monitored as the occurring climate change is considered as a potential factor influencing tick abundance as well as distribution of tick-borne pathogens [25, 26]. However, consistently performed long-term monitorings on Rickettsiales in questing ticks are still lacking. Thus, this first 10-year follow-up monitoring conducted with largely identical study design in the city of Hanover, Germany, aimed to address the question whether tick infection rates with Rickettsiales are stagnating, decreasing or increasing over years.

## Methods

### Tick material, sampling sites and climate data

From April to October 2015, questing *I. ricinus* ticks were collected monthly by the flagging method using a white linen fabric sheet (approximately 120 × 60 cm) that was slowly drawn in meandering shape over the ground. Flagging was conducted until 30 ticks per location/month were collected. Collection was performed on rain-free days at ten different recreation areas frequently visited by residents and tourists in the city of Hanover. The capital of the northern German federal state Lower-Saxony is well known as a city with numerous city parks, gardens and urban woodland serving as a natural habitat for a large variety of rodents and birds. The study design was based on previous studies conducted in 2005 and 2010 in Hannover [8, 9, 12, 19] and served as a 10-year follow-up monitoring, although two sampling sites differed in 2010 and 2015 compared to 2005 due to organizational or construction measures. A total of 30 ticks were collected and analysed per location and month resulting in a total of 2100 investigated ticks. The ticks were identified according to the morphological key by Estrada-Peña et al. [27] and individually stored in sterile tubes at −20 °C until genomic DNA isolation.

### Genomic DNA isolation

Ticks were individually homogenized with polystyrene pistils (Carl Roth GmbH, Karlsruhe, Germany) and genomic DNA was isolated using the Nucleo spin® 8 Blood Kit (Macherey-Nagel, Düren, Germany) according to the manufacturer’s instructions with previously described modifications [8, 13]. Isolated genomic DNA was stored at -20 °C until further use.

### Quantitative real-time PCR

A duplex qPCR targeting the *msp2*/*p44* gene based on a primer-probe combination by Courtney et al. [28] was performed to detect *A. phagocytophilum* as described previously [8]. In addition to *A. phagocytophilum* detection, successful DNA isolation was verified by targeting the *I. ricinus* ITS2 region [29]. Reaction set-ups and thermal cycling were performed as described by Tappe et al. [8] except that the amount of tick DNA template was increased to 10 μl.

The citrate synthase (*gltA*) gene served as the target sequence for detection of *Rickettsia* spp. using a primer-probe combination described by Stenos et al. [30]. The reactions and thermal cycling were carried out as published previously [8].

### Real-time pyrosequencing for determination of *Rickettsia* spp.

A subset of 250 *Rickettsia* spp.-positive ticks was identified for the infecting *Rickettsia* species using real-time pyrosequencing as described previously [8, 9, 13, 31].

### Statistical analyses

Data were statistically analysed by Chi-square test with subsequent Bonferroni-Holm correction. Fisher’s exact test was applied to analyse *A. phagocytophilum* data due to low prevalences. Analyses comprised comparison of tick stages (nymphs, adult females, adult males and total ticks), sampling months and sampling sites in 2015 as well as comparison with previous data obtained in 2010 and 2005. In 2005, sampling months were partially combined for statistical analyses of *A. phagocytophilum* infection rates. Furthermore, infection rates were determined starting in March, whereas studies in 2010 and 2015 started in April. As data of individual sampling months were not available for 2005, data obtained in 2010 and 2015 were modified accordingly to allow statistical comparison of seasonal data. Due to variations of two sampling sites between 2005 and the follow-up studies conducted in 2010 and 2015, comparison of local distribution of *A. phagocytophilum* infection rates was not feasible. As data on *Rickettsia* infection rates obtained in 2005 were not evenly distributed over the sampling months as well as sampling sites, seasonal and local distribution could only be compared for 2010 and 2015. All statistical analyses were performed with the GraphPad Prism™ software (version 6.03, La Jolla, CA, USA).

## Results

### Tick material and genomic DNA isolation

Morphological species examination by macroscopic identifiable parameters classified all collected ticks as *I. ricinus*. Determination of stage as well as sex resulted in 573 adults (288 females and 285 males) in addition to 1527 nymphs.

### Tick infections with *A. phagocytophilum* in 2015

The overall prevalence of *A. phagocytophilum* was 3.8% (79/2100). Adult ticks showed an infection rate of 7.2% (41/573) subdivided into 9.4% (27/288) infected females and 4.9% (14/285) males. Both, males and females, were significantly more frequently infected than nymphs (2.4%; 38/1527) (Fisher’s exact test, *P* = 0.0324 and *P* < 0.0001, respectively). A peak in tick infections was observed in July (12.0%; 36/300) which differed significantly from the remaining sampling months (Fisher’s exact test, *P* < 0.0001). Detailed information about seasonal distribution of infected tick stages is provided in Table 1.

**Table 1:** Seasonal distribution of *A. phagocytophilum* and *Rickettsia* spp. infected ticks collected in Hanover, Germany, in 2015

	April	May	June	July	August	September	October	Total
*Anaplasma phagocytophilum* ^a^								
Adults	4/95 (4.2)	5/97 (5.2)	6/95 (6.3)	16/80 (20.0)	2/75 (2.7)	2/72 (2.8)	6/59 (10.2)	41/573 (7.2)*
Adult males	2/53 (3.7)	3/48 (6.3)	2/51 (3.9)	5/42 (11.9)	1/37 (2.7)	0/28 (0)	1/27 (3.7)	14/285 (4.9)*
Adult females	2/43 (4.7)	2/49 (4.1)	4/44 (9.1)	11/38 (28.9)	1/38 (2.6)	2/44 (4.5)	5/32 (15.6)	27/288 (9.4)*
Nymphs	0/205 (0)	0/203 (0)	3/205 (1.5)	20/220 (9.1)	4/225 (1.8)	3/228 (1.3)	8/241 (3.3)	38/1527 (2.4)*
Total	4/300 (1.3)^§^	5/300 (1.7)^§^	9/300 (3.0)^§^	36/300 (12.0)^§^	6/300 (2.0)^§^	5/300 (1.7)^§^	14/300 (4.7)^§^	79/2100 (3.8)
*Rickettsia* spp.^a^								
Adults	33/95 (34.7)	47/97 (48.5)	35/95 (36.8)	55/80 (68.8)	53/75 (70.7)	50/72 (69.4)	37/59 (62.7)	310/573 (54.1)
Adult males	18/52 (34.6)	24/48 (50.0)	18/51 (35.3)	28/42 (66.7)	23/37 (62.2)	21/28 (75.0)	12/27 (44.4)	144/285 (50.5)
Adult females	15/43 (34.8)	23/49 (46.9)	17/44 (38.6)	27/38 (71.1)	30/38 (78.9)	29/44 (65.9)	25/32 (78.1)	166/288 (57.6)**
Nymphs	79/205 (38.5)	81/203 (39.9)	71/205 (34.6)	133/220 (60.4)	138/225 (61.3)	129/228 (56.6)	125/241 (51.9)	756/1527 (49.5)**
Total	112/300 (37.3)^§§^	128/300 (42.7)^§§^	106/300 (35.5)^§§^	188/300 (62.7)^§§^	191/300 (63.7)^§§^	179/300 (59.7)^§§^	162/300 (54.0)^§§^	1066/2100 (50.8)

^a^No. of positive ticks/ Total no. of ticks (%)

*Significantly higher infection rates in adult ticks (females and males) vs nymphs (*P* ≤ 0.05)

**Significantly higher infection rates in adult females vs nymphs (*P* ≤ 0.05)

^§^Significantly higher infection rates in July vs April, May, June, September and October (*P* ≤ 0.05)

^§§^Significantly higher infection rates in July, August and September vs April, May and June as well as in October vs April and June (*P* ≤ 0.05)

Concerning sampling sites, statistically significant differences were detected between “Misburger Wald” (9.1%; 19/210) representing the location with the highest prevalence of *A. phagocytophilum* infections vs “Annateiche” (1.0%; 2/210; Fisher’s exact test, *P* = 0.0002) and “Mecklenheide” (0.0%; 0/210; Fisher’s exact test, *P* < 0.0001), the locations with the lowest infection rates. Furthermore, significant differences between the locations “Eilenriede” (6.7%; 14/210; Fisher’s exact test, *P* < 0.0001) and “Bornumer Holz” (5.7%; 12/210, Fisher’s exact test, *P* = 0.0004) vs “Mecklenheide” (0.0%; 0/210) were determined. Detailed data regarding tick infection rates at the different sampling sites are provided Table 2.

**Table 2:** Local distribution of *A. phagocytophilum* and *Rickettsia* spp. infected ticks collected in Hanover, Germany, in 2015

	Mecklenheide	Große Heide	Misburger Wald	Annateiche	Seelhorster Wald	Ricklinger Teiche	Bornumer Holz	Georgengarten	Eilenriede	Maschpark
*Anaplasma phagocytophilum* ^a^										
Adults	0/72 (0)	4/84 (4.8)	1/20 (5.0)	1/40 (2.5)	7/93 (7.5)	0/40 (0)	10/74 (13.5)	3/36 (8.3)	11/91 (12.1)	3/23 (13.0)
Adult males	0/35 (0)	1/37 (2.7)	1/11 (9.1)	1/21 (4.8)	2/45 (4.4)	0/23 (0)	2/39 (5.1)	0/19 (0)	7/46 (15.2)	0/9 (0)
Adult females	0/37 (0)	3/47 (6.4)	0/9 (0)	0/19 (0)	5/48 (10.4)	0/17 (0)	8/35 (22.9)	3/17 (17.6)	4/45 (8.9)	3/14 (21.4)
Nymphs	0/138 (0)	0/126 (0)	18/190 (9.5)	1/170 (0.6)	1/117 (0.6)	6/170 (3.5)	2/136 (1.5)	0/174 (0)	3/119 (2.5)	3/187 (1.6)
Total	0/210 (0)*	4/210 (1.9)	19/210 (9.1)*	2/210 (1.0)*	8/210 (3.8*)	6/210 (2.9)	12/210 (5.7)*	8/210 (3.8)	14/210 (6.7)*	6/210 (2.9)
*Rickettsia* spp.^a^										
Adults	37/72 (51.4)	41/84 (48.8)	15/20 (75.0)	25/40 (62.5)	44/93 (47.3)	22/40 (55.0)	44/74 (59.5)	16/36 (44.4)	52/91 (57.1)	14/23 (60.9)
Adult males	17/35 (48.6)	16/37 (43.2)	8/11 (72.7)	9/21 (42.9)	18/45 (40.0)	13/23 (56.5)	22/39 (56.4)	7/19 (36.8)	27/46 (58.7)	7/9 (77.8)
Adult females	20/37 (54.1)	25/47 (53.2)	7/9 (77.8)	16/19 (84.2)	26/48 (54.1)	9/17 (35.3)	22/35 (62.9)	9/17 (52.9)	25/45 (55.6)	7/14 (50.0)
Nymphs	55/138 (39.9)	51/126 (40.5)	112/190 (58.9)	88/170 (51.8)	45/117 (38.5)	93/170 (54.7)	65/136 (47.8)	82/174 (47.1)	64/119 (53.8)	101/187 (54.0)
Total	92/210 (43.8)**	92/210 (43.8)**	127/210 (64.3)**	113/210 (53.8)	89/210 (42.4)**	115/210 (54.8)	109/210 (51.9)	98/210 (46.7)	116/210 (55.2)	115/210 (54.8)

^a^No. of positive ticks/ Total no. of ticks (%)

*Significantly higher infection rates in “Misburger Wald”, “Bornumer Holz” and “Eilenriede” vs “Mecklenheide”, as well as “Misburger Wald” vs “Annateiche” (*P* ≤ 0.05)

**Significantly higher infection rates in “Misburger Wald” vs “Mecklenheide”, “Große Heide” and “Seelhorster Wald” (*P* ≤ 0.05)

### Tick infections with *A. phagocytophilum* in 2015 vs 2010 and 2005

**Fig. 1:**
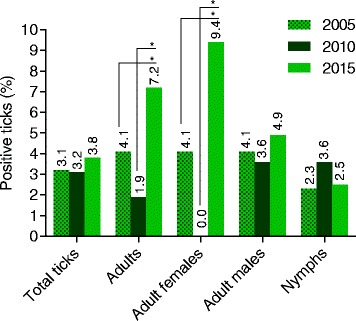
Stadial distribution of *A. phagocytophilum-*positive ticks studied in Hanover during 2005–2015 [8, 9, 12]. *P ≤ 0.05

Over the entire monitoring period of 10 years, total tick infection rates with *A. phagocytophilum* remained constant; however, statistically significant differences between tick stages were observed. In 2015, adult ticks were significantly more often infected than ten years before [7.2 vs 4.1% in 2005 (*P* = 0.0205) and 1.9% in 2010 (*P* = 0.0005); Fig. 1] [8, 9, 12]. Furthermore, adult females showed significantly higher infection rates in 2015 (9.4%; 27/288) compared to 2010 (0.0%; 0/176; *P* < 0.0001) and 2005 (4.1%; 16/388; *P* = 0.0091) [12].

**Fig. 2:**
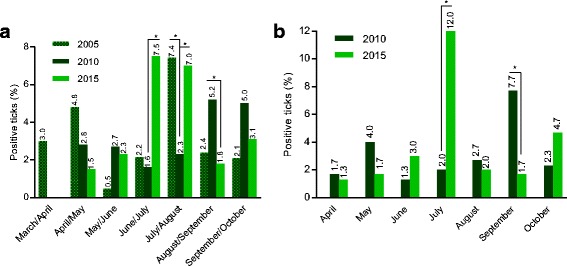
Seasonal distribution of *A. phagocytophilum*-positive ticks studied in Hanover. **a** 2005–2015 (as data of individual sampling months was not available for 2005, data obtained in 2010 and 2015 were modified accordingly) [8, 9, 12]. **b** 2010 [8, 9] vs 2015. *P ≤ 0.01

Regarding seasonal distribution of *A. phagocytophilum*, statistically significant differences between June/July 2015 (7.5%) vs June/July 2010 (1.6%) [8, 9] were determined (Fisher’s exact test, *P* < 0.0001). Further significant differences were observed between July/August in years 2015 (7.0%) and 2005 (7.4%) [12] vs July/August 2010 (2.3%) (Fisher’s exact test, *P* = 0.0002 and *P* = 0.0013, respectively) [8, 9]. By contrast, in August/September 2010 (5.2%) as well as September 2010 (7.7%) [8, 9] significantly higher *A. phagocytophilum* infection rates were noted compared to August/September 2015 (1.8%) and September 2015 (1.7%) (Fisher’s exact test, *P* = 0.0024 and *P* = 0.0007, respectively). More detailed results of seasonal analyses are provided in Fig. 2. Sampling site distributions of *A. phagocytophilum* infected ticks did not show significant differences between 2010 and 2015.

### Tick infections with *Rickettsia* spp. in 2015

In 2015, the overall prevalence of infection with *Rickettsia* spp. was 50.8% (1066/2100). Subdivided into stages, 54.1% (310/573) of adult ticks tested positive, thereof 57.6% (166/288) females and 50.5% (144/285) males. Female ticks were found to be more frequently infected than nymphs (49.5%; 756/1527; *χ* ^2^ = 6.087, *df* = 1, *P* = 0.0136). Furthermore, significant seasonal variations in infection rates were determined between the first three (April to June) and the last four sampling months (July to October; *χ* ^2^ = 16.13–47.04, *df* = 1, *P* ≤ 0.0001), with exception of the comparison May vs October (Fig. 1). Detailed seasonal infection rates are provided in Table 1. Referring to sampling sites, significantly higher infection rates were determined for “Misburger Wald” (64.3%; 127/210), the location with the highest infection rate, vs “Mecklenheide”/ “Große Heide” (43.8% each; 92/210; *χ* ^2^ = 11.03, *df* = 1, *P* = 0.0009) and “Seelhorster Wald” (42.4%; 89/210; *χ* ^2^ = 13.05, *df* = 1, *P* = 0.0003). Table 2 provides detailed data about local *Rickettsia* spp. distribution in ticks in Hanover.

### Tick infections with *Rickettsia* spp. in 2015 vs 2010 and 2005

**Fig. 3:**
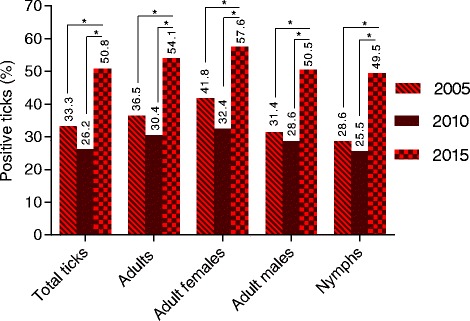
Stadial distribution of *Rickettsia*-positive ticks studied in Hanover during 2005–2015 [8, 19]. *P ≤ 0.05

**Fig. 4:**
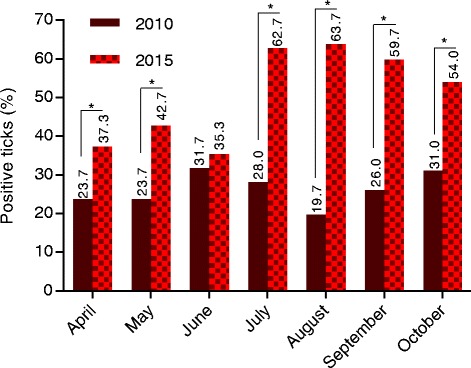
Seasonal distribution of *Rickettsia*-positive ticks studied in Hanover during 2010–2015 [8, 19]. *P ≤ 0.001

Significantly increased *Rickettsia* spp. infection rates were determined over the 10-year monitoring period. Total prevalence increased from 33.3% in 2005 [19] to 50.8% in 2015 with all tick stages showing increased *Rickettsia* infection rates in 2015 compared to 2010 and 2005 (*χ* ^2^ = 14.38–197.7, *df* = 1, *P* < 0.0001; Fig. 3). Significantly increased infection rates were also observed regarding seasonal variations. In 2015, all months but June showed higher *Rickettsia* infection rates than in 2010 [8] (*χ* ^2^ = 23.58–117.7, *df* = 1, *P* < 0.0001; except April 2015 vs April 2010: *χ* ^2^ = 12.58, *df* = 1, *P* = 0.0004; Fig. 4). Unfortunately, seasonal comparison to 2005 was not possible (cf. Methods). Analysis of the sampling site-associated infections resulted in significantly increased prevalences for each location in 2015 compared to 2010 (*χ* ^2^ = 18.66–43.56, *df* = 1, *P* < 0.0001) for each location, except for “Seelhorster Wald” (*χ* ^2^ = 10.09, *df* = 1, *P* = 0.0015) and “Maschpark” (*χ* ^2^ = 13.87, *df* = 1, *P* = 0.0002).

### Real-time pyrosequencing for identification of *Rickettsia* spp.

Real-time pyrosequencing successfully identified the infecting *Rickettsia* spp. in 158 of the 250 analysed ticks (63.2%). Of these, 99.4% (157/158) were infected with *R. helvetica* and 0.6% (1/158) with *R. monacensis.* Regarding real-time pyrosequencing sensitivity, samples containing ≥ 10^5^ *gltA* copies as determined in qPCR were identified in 100% of the samples (1/1), the range of 10^4^ copies in 90.1% (73/81), 10^3^ in 88.9% (72/81), 10^2^ in 100% (2/2) and 10^1^ in 40% (4/10). In samples containing less than 10 gene copies, species identification succeeded in 8.0% (6/75).

### Coinfections in ticks with Rickettsiales in 2015 vs 2010 and 2005

In 2015, 5.2% (15/288) of females, 2.4% (7/285) of males and 1.6% (24/1527) of nymphs showed coinfections with both pathogens, resulting in a total of 2.2% (46/2100) co-infected ticks. This is significantly higher (*χ* ^2^ = 17.60, *df* = 1, *P* < 0.0001) than the total coinfection rate of 0.6% reported in 2010 [7, 10], but similar to 2.8% determined in 2005 [19]. Regarding tick stages, adult females were significantly more often co-infected in 2015 compared to 2010 (*χ* ^2^ = 7.881, *df* = 1, *P* = 0.005), whereas comparison of co-infected stages between 2015 and 2005 revealed no significant differences.

## Discussion

Rickettsiales (*A. phagocytophilum* and *Rickettsia* spp.) transmitted by ticks may be a threat to human and animal welfare*.* Several studies determined tick infection rates with named pathogenic microorganisms [1, 8–13, 19]; however, periodically performed long-term monitoring studies based on a comparable set-up are scarce, even though such data are needed for a continuous public health risk assessment. The presented study conducted in the northern German city of Hanover is the first German 10-year follow-up monitoring on tick infections rates with Rickettsiales.

In 2015, almost every 25th tick (3.8%) was infected with *A. phagocytophilum*. Our results are consistent with the data reported in recent surveys, which described *A. phagocytophilum* prevalences to vary between 0% in central Germany [10] and 9.5% in southern Germany [11]. Over the monitoring period of ten years, the overall *A. phagocytophilum* prevalence remained constant in the city of Hanover. However, a significant increase of infection rates in adult ticks was observed over the course of ten years, which is mostly attributed to female tick infection rates as solely females showed significantly higher infection rates in 2015 (9.4%) compared to 2005 (4.1%) and 2010 (0.0%) [8, 9, 12]. It may be speculated that the observed increase of infected adult ticks may be due to a more efficient transstadial transmission of *A. phagocytophilum* [3, 32] in recent years, but also to higher numbers of collected females at particular sampling sites. Interestingly, highest overall infection rates in the presented study years were ascribed to three sampling sites, all representing forest areas. However, vegetation alone is not representative for high *A. phagocytophilum* infections in ticks, but rather high numbers of infected reservoir hosts. For example, at the forest sampling site “Mecklenheide”, no ticks were determined *A. phagocytophilum*-positive in contrast to the study maximum of 9.1% infected ticks at the forest site “Misburger Wald”. These differences in infection rates at locations with comparable vegetation are in accordance with findings of several previously conducted surveys [8, 9, 12, 13]. Regarding infected host availability, Overzier et al. [33] detected *Anaplasma* DNA in 98.9% of investigated roe deer as well as in 86.1% of engorged adult ticks. Simultaneously, 8.9% of questing adult ticks in the study area were determined *A. phagocytophilum-*positive, indicating a correlation between infected roe deer as reservoir hosts and infected ticks. Regarding the potential human pathogenicity, roe deer has been described as preferred host for *A. phagocytophilum* strains with low human pathogenicity [4]. Contrary, strains associated with human, canine and equine anaplasmosis were mainly found in wild boars and hedgehogs [4, 34, 35]. In Germany, about 14.0% of tick-exposed individuals like hunters, forestry workers or farm workers were tested positive for antibodies against *A. phagocytophilum* [36], indicating frequent human infection after exposure to infected ticks [36]. It is likely that due to high infection rates in roe deer [33] many circulating *A. phagocytophilum* strains in Germany are deer-associated and therefore presumably non-pathogenic to humans [4, 37]. This could also be a reason for the discrepancy between the particularly high seroprevalences and contrary low numbers of HGA cases across Europe [4, 37]. Additionally, mild or unspecific symptoms may contribute to possibly lacking recognition of HGA by clinicians [37].

The seasonal distribution of *A. phagocytophilum* was characterized each sampling year by main peaks of infection rates in July 2015, July/August 2005 [12] and September 2010 [8, 9], which indicates recurrent peaks of tick infections with the beginning or progression of the second half of tick activity season [38]. Peaking tick infection rates may correspond to a peak in available infected reservoir hosts one or two years prior the respective sampling years, in which ticks infected by feeding on these reservoir hosts were collected as new questing ticks after moulting to the next stage. In addition to presumably increased reservoir host activity, acquired infections may also have increased due to high amounts of co-feeding ticks on infected vertebrates [39]. The observed differences in the respective “peak months” between sampling years also explain the observed seasonal differences in infected ticks over the 10-year study period. In contrast, results of a comparable study conducted in the city of Hamburg, Germany, showed consistent *A. phagocytophilum* tick infection rates during the sampling period from April to October [13]. However, as tick infection data originating from the city of Hamburg is solely available for the year 2011, it may not be determined whether the lacking infection peak is a stable epidemiological finding at this location or an arbitrary observation, especially as total annual prevalences in both cities are comparable.

Contrary to consistent overall *A. phagocytophilum* infection rates, significantly increasing tick infection rates with *Rickettsia* spp. over the 10-year monitoring period were detected with approximately every second tick being infected in 2015. More precisely, the significant increase in infection rates was determined between 2010 and 2015 and concerned all collected tick stages, i.e. nymphs as well as adult male and female ticks. Species identification confirmed *R. helvetica* as the predominant species and a sporadic presence of *R. monacensis* as observed in previous studies conducted in Germany [8, 9, 13, 19]. Interestingly, the so far determined *R. monacensis-*positive ticks, found in 2005 and 2015, originated from in the Hanover district “Ricklingen”, possibly indicating a small local endemic population. As species distribution appears consistent, simultaneous function of ticks as vectors, main hosts and reservoir hosts for *Rickettsia* has to be taken into consideration to elucidate increased prevalences. In this regard, highly efficient transovarial transmission of up to 100% in *I. ricinus* under laboratory conditions chiefly contributes to the maintenance of the *Rickettsia* life-cycle [17, 40–43]. Even if the efficacy of transovarial transmission in the field would be significantly lower, it ultimately contributes to an increase of *Rickettsia* infections in ticks. This accumulation will probably be enhanced by transmission from infected males to females through spermatids, spermatophores, and spermiophore fluids as reported for *R. helvetica* [44, 45], as well as feeding of the ticks on infected hosts. As development from larvae to adult ticks usually requires several years [38], the observed significant increase in tick *Rickettsia* infections has occurred prior to collection of questing nymph and adult ticks in 2015. Nevertheless, bacterial infection may negatively affect infected ticks as bacterial overload and direct lethal effects of bacterial infection on ticks have been described [46, 47]. Consequently, these effects may aid in elucidating determined variations between study years and may have caused lower infection rates in the years 2005 and 2010. It remains unknown whether the significant increase in questing ticks occurred only in study year 2015 or already between 2011 and 2014 as ticks were only examined every 5 years. Overall, the observed total *Rickettsia* prevalence of 50.8% is considerably higher than in other German regions. In central Germany and at the German Baltic coast, only 4.1% and 8.5% of ticks, respectively, were determined *Rickettsia-*positive [10, 23]. Considerably higher prevalences of 33.3% were previously determined in the city of Hanover [19] in addition to Hamburg, which is represented by the highest *Rickettsia* prevalence in ticks (52.5%) [13].

The differences in tick *Rickettsia* infections at different sampling locations detected in our study are in accordance with previous data obtained in the city of Hanover [8, 19]. As observed for *A. phagocytophilum*, the lowest *Rickettsia* tick infection rate was found at the forest sampling site “Mecklenheide”, whereas the forest site “Misburger Wald” represented the location with the highest infection rate. Interestingly, *Rickettsia* prevalences showed prominent seasonality in the current study, resulting in significantly lower number of infected ticks in April–June compared to July–October. Similarly, distinct seasonality was also recorded in the city of Hamburg [13], with infection rates significantly increasing starting in June, one month earlier than in Hanover. This seasonality may also depend on the distribution of *Rickettsia* spp. in ticks as well as on the abundance of potential vertebrate reservoir hosts, which are thought to be mainly constituted by small mammals [17, 41, 43]. It has been hypothesised that *Rickettsia* infections are persistent in ticks serving as vectors, but transient in mammals as suspected reservoir hosts [41, 43], which possibly eliminate *Rickettsia* infections during winter. This may contribute to the observed seasonality in terms of significantly increased *Rickettsia* tick infection rates during July–October compared to April–June. Interestingly, examination of trapped wild mice has demonstrated an accumulation of *Rickettsia* spp. in ear tissues, indicating a mostly local *Rickettsia* infection in vertebrates. A large proportion of attached larvae and nymphs was feeding on ears of trapped mice resulting in a high infection potential during the blood meal [41], enabling efficient transmission cycles between ticks and the small mammal host. In this context, the possibility of co-feeding as another way of pathogen transmission should be considered [39].

Regarding tick co-infections, the significantly increased tick *Rickettsia* infections in 2015 may also account for significantly higher occurrence of co-infections with *A. phagocytophilum* compared to the study in 2010 [8, 9]. However, despite consistent *A. phagocytophilum* infection rates over the 10-year monitoring period, no significant differences in co-infection rates between 2015 and 2005 [19] were observed. Furthermore, co-infection rates of 2015 and 2005 [19] are in accordance with those obtained by May & Strube [13] in 2011 within the city of Hamburg.

## Conclusions

Overall, tick infection rates with *A. phagocytophilum* remained consistent over the monitoring period of ten years, whereas a significant increase of *Rickettsia* infected ticks was observed. To date, the city of Hamburg represents the area with the highest determined *Rickettsia* infection rate in Germany [13]. Nevertheless, comparably high *Rickettsia* prevalences were detected in the present study in Hanover, which is located about 150 km further south. The approximately doubled *Rickettsia* prevalence in ticks in Hanover compared to previous years [8, 19] also poses an increased risk to public health. Consequently, monitoring of human disease would contribute to defining a potential correlation of increasing tick infection rates and patients suffering from *Rickettsia* infection after tick exposure. Based on the assumption of a relevantly transovarial-driven *Rickettsia* infection dynamics in ticks, a further continuous growth is to be expected. A future study in 2020 will provide further long-term data on the dynamics of Rickettsiales infections in ticks in Hanover.

## Abbreviations

HGA: human granulocytic anaplasmosis
